# MHC Class II Activation and Interferon-γ Mediate the Inhibition of Neutrophils and Eosinophils by Staphylococcal Enterotoxin Type A (SEA)

**DOI:** 10.3389/fcimb.2017.00518

**Published:** 2017-12-13

**Authors:** Ana P. Ferreira-Duarte, Anelize S. Pinheiro-Torres, Gabriel F. Anhê, Antônio Condino-Neto, Edson Antunes, Ivani A. DeSouza

**Affiliations:** ^1^Department of Biology and Physiology, Faculty of Medicine of Jundiai, Jundiai, Brazil; ^2^Department of Pharmacology, State University of Campinas, Campinas, Brazil; ^3^Department of Immunology, Institute of Biomedical Sciences, University of São Paulo, São Paulo, Brazil

**Keywords:** adhesion, chemotaxis, bone marrow, interleukin-8, eotaxin

## Abstract

Staphylococcal enterotoxins are classified as superantigens that act by linking T-cell receptor with MHC class II molecules, which are expressed on classical antigen-presenting cells (APC). Evidence shows that MHC class II is also expressed in neutrophils and eosinophils. This study aimed to investigate the role of MHC class II and IFN-γ on chemotactic and adhesion properties of neutrophils and eosinophils after incubation with SEA. Bone marrow (BM) cells obtained from BALB/c mice were resuspended in culture medium, and incubated with SEA (3–30 ng/ml; 1–4 h), after which chemotaxis and adhesion were evaluated. Incubation with SEA significantly reduced the chemotactic and adhesive responses in BM neutrophils activated with IL-8 (200 ng/ml). Likewise, SEA significantly reduced the chemotactic and adhesive responses of BM eosinophils activated with eotaxin (300 ng/ml). The inhibitory effects of SEA on cell chemotaxis and adhesion were fully prevented by prior incubation with an anti-MHC class II blocking antibody (2 μg/ml). SEA also significantly reduced the intracellular Ca^2+^ levels in IL-8- and eotaxin-activated BM cells. No alterations of MAC-1, VLA4, and LFA-1α expressions were observed after SEA incubation. In addition, SEA elevated by 3.5-fold (*P* < 0.05) the INF-γ levels in BM cells. Incubation of BM leukocytes with IFN-γ (10 ng/ml, 2 h) reduced both neutrophil and eosinophil chemotaxis and adhesion, which were prevented by prior incubation with anti-MHC class II antibody (2 μg/ml). In conclusion, SEA inhibits neutrophil and eosinophil by MHC class II-dependent mechanism, which may be modulated by concomitant release of IFN-γ.

## Introduction

*Staphylococcus aureus* is one of the most important human pathogen associated with severe hospital-acquired infections, including pneumonia, endocarditis, and sepsis (Adhikari et al., 2012; Nair et al., 2014). *Staphylococcus aureus* infections have been strongly associated to its ability to produce several virulent factors such as adhesins, collagenases, protein A, coagulases, hemolysins, and leukocidins (Krakauer and Stiles, 2013). *Staphylococcus aureus* also produce the staphylococcal enterotoxins, which are a family of structurally related heat-stable 25–30 kDa proteins, comprising five major serological types (SEA to SEE) (Ono et al., 2015) and new types of SE-related toxins (SEG to SElZ) (Spoor et al., 2015). In animal models, exposure to Staphylococcal enterotoxin types A (SEA) and B (SEB) induces acute lung injury characterized by a marked granulocyte infiltration and production of pro-inflammatory mediators, which results in reduced lung function (Herz et al., 1999; DeSouza et al., 2005; Desouza et al., 2006; Hellings et al., 2006; Mariano et al., 2010; Rao et al., 2015). Polyclonal and monoclonal antibodies against staphylococcal enterotoxin**s** (SEA and SEB) prevent *S. aureus*-induced sepsis and inflammation, as well as animal mortality (Hobeika and Johnson, 1996; Varshney et al., 2013; Xia et al., 2014).

Staphylococcal enterotoxins are superantigens due its properties to induce extensive proliferation of T cells mediated by cross-linking of the variable region of the β chain of the T-cell receptor (TCR) with MHC class II molecules on antigen-presenting cells (APC) such as macrophages and dendritic cells (Fraser, 2011). MHC molecules are heterodimeric cell-surface glycoproteins constitutively expressed on APC surface (Roche and Furuta, 2015). MHC class II molecules function to present antigen to CD4 T lymphocytes generating helper T cell responses, which are critical for effective adaptive immune responses against infection (Afridi et al., 2016). Human and murine neutrophils (Ostanin et al., 2012; Pliyev et al., 2014) and eosinophils also express MHC Class II (Wang et al., 2007; Farhan et al., 2016), but little is known about the role of this glycoprotein in these granulocytes.

Infection conditions are frequently accompanied by mature immune cell depletion, and require a rapid bone marrow (BM) compensatory response to reestablish the cellular homeostasis in blood (Furze and Rankin, 2008). Interferon γ (IFN-γ) is a cytokine implicated in innate and acquired immune response during bacterial infections (Eshleman and Lenz, 2014). IFN-γ is typically produced by natural killer T cells during an immune response to intracellular pathogens, like mycobacteria and viruses. Additionally, IFN-γ exerts an important role on the proliferation and differentiation of bone marrow (BM) myeloid cells, but high levels of this cytokine are suggested to suppress the hematopoiesis, leading to leukopenia that is clinically observed after the resolution of severe infectious conditions (Baldridge et al., 2010; de Bruin et al., 2014). IFN-γ is also well-characterized to induce endothelial cell adhesion molecule overexpression and to promote leukocyte adhesion and recruitment (Zhang et al., 2011), but display an inhibitory effect on Th2 responses and eosinophilia (Park et al., 2005; Kanda et al., 2009). Moreover, IFN-γ release is reported to contribute to pathological events induced by Staphylococcal enterotoxin in mice (Plaza et al., 2007; Muralimohan et al., 2008; Kuroda-Morimoto et al., 2010; Rao et al., 2014). Evidence shows that IFN-γ induces overexpression of MHC class II in human circulating neutrophils (Pliyev et al., 2014) and reduces *S. aureus*-mouse induced neutrophil infiltration (Barin et al., 2016). IFN-γ also induces synthesis and expression of MHC class II by blood-derived human eosinophils (Weller et al., 1993; Shi, 2004). These findings support the hypothesis that IFN-γ, via MHC class II activation, modulates the inhibitory effects of Staphylococcal enterotoxins in granulocytes.

A recent study has showed that SEA inhibits the activity of eosinophils (Squebola-Cola et al., 2014). Therefore, in the present study, we designed experiments to investigate the mechanisms by which SEA inhibits the activity of leukocytes. Specifically, we have used mouse BM leukocytes to explore the role of MHC class II and IFN-γ in mediating the inhibitory effects of SEA on the *in vitro* adhesion and chemotaxis activated with interleukin-8 (neutrophils) and eotaxin (eosinophils).

## Materials and methods

### Materials

Staphylococcal enterotoxin A (SEA), eotaxin and ethylene glycol-bis (β-aminoethyl ether)-N,N,N′,N′ tetraacetic acid (EGTA) were purchased from Sigma Aldrich Co (St. Louis, MO, USA). Iscove's modified Dulbecco's medium (IMDM) was obtained from life technologies (New York, USA). ELISA kits for mouse IFN-γ, vascular cell adhesion molecule-1 (VCAM-1), intercellular adhesion molecule 1 (ICAM-1), fluorescein isothiocyanate-conjugated anti-mouse MAC-1, phycoerythrin (PE)-conjugated anti-mouse VLA-4, fluorescein isothiocyanate-conjugated anti-mouse LFA1-α and interleukin-8 (IL-8) were obtained from BD Biosciences Pharmingen (San Jose, CA, USA). Fluoforte was obtained from Enzo Life Sciences International (New York, USA). Interferon-γ was purchased from Boehringer Ingelheim (Berkshire, UK). Antibody anti-MHC class II was obtained from Abcam (Cambridge, UK).

### Animal experimentation guidelines

All animal care and experimental protocols were approved by the Ethical Principles in Animal Research adopted by the Brazilian College for Animal Experimentation (COBEA), and followed the Guide for the Care and Use of Laboratory Animals. Four-week-old male BALB/c mice were provided by the Central Animal House Services of State University of Campinas (UNICAMP). Animals were housed three per cage on a 12 h light–dark cycle, in temperature-controlled rooms and received water and food *ad libitum* until used on the Animal House Services of Faculty of Medicine of Jundiaí (FMJ).

### Bone marrow (BM) collection and isolation of granulocytes

Mouse bone marrow (BM) granulocyte isolation was carried out according to a previous study (Lintomen et al., 2002). Briefly, BM cells were collected and subsequently placed in plates (100 × 20 mm dish) for 30 min at 37°C (5% CO_2_). The BM supernatants (non-adhered cells) were collected, washed twice with 2 ml of Iscove's modified Dulbecco's culture medium, and centrifuged (500 g for 10 min at 4°C). The non-adherent BM cell pellets were resuspended in 2.5 ml of culture medium, and the total (Neubauer) and differential (Diff-Quick stain) cell counts were done. The final cell suspension contained about of 70% of mature granulocytes, consisting of 60% neutrophils and 10% eosinophils, whereas the remainder was made of 30% of mononuclear cells. The adhesion and chemotaxis of neutrophils and eosinophils were evaluated through the measurements of specific peroxidases for neutrophils (myeloperoxidase; MPO) and eosinophils (eosinophil peroxidase; EPO), as detailed below.

### Incubation of BM cells with sea or IFN-γ

Cells incubated with SEA (1–30 ng/ml), IFN-γ (10 ng/ml) or sterile IMDM (control group) at 37°C, 5% CO_2_ for 30 min to 4 h, after which chemotaxis, adhesion, Ca^2+^ levels and integrin expression were evaluated, as detailed below. In some assays, cells were pretreated with MHC class II blocking antibody, a detailed below.

### Neutrophil adhesion and chemotaxis assays

The number of neutrophils in BM was initially adjusted to 4 × 10^6^ cells/ml. Next, BM cells were incubated with SEA (1–30 ng/ml), IFN-γ (10 ng/ml) or sterile IMDM (*control* group) at 37°C, 5% CO_2_ for 30 min to 4 h. Adhesion and chemotaxis assays for neutrophils were performed as described below. For the adhesion assays, 50 μL of BM cells, treated or not with SEA, were added to 96-well plates pre-coated with recombinant mouse VCAM-1 (2.5 μg/ml) or ICAM-1 (2.5 μg/ml) (Takeshita et al., 2015)_._ BM neutrophils were then stimulated with IL-8 (300 ng/ml) for 30 min, and adhesion was calculated by measuring the myeloperoxidase (MPO) activity of adherent cells. Briefly, each well received hexadecyltrimethyl ammonium bromide (HTBA) 0.5% in 50 mM of potassium phosphate buffer, pH 6.0. Thereafter, each well received 200 μl of *o*-dianisidine solution (*o*-dianisidine: 0.167 mg/ml; hydrogen peroxide: 0.0005% in 50 mM of phosphate buffer, pH 6.0) immediately before absorbance measurement at 460 nm with a microplate reader (Synergy H1 Hybrid Reader, Biotek, USA). Neutrophil adherence was calculated using the optic density (OD) obtained in MPO assay divided by the number of neutrophils (NE) added in each well.

For the chemotaxis assays, we used a 96-multiwell chemotaxis chamber (Bignold et al., 1990). The wells in the microplate at the bottom compartment were filled with 29 μl of IL-8 (200 ng/ml). A polycarbonate filter (5 μm pore size) was positioned on the loaded microplate and hold in place with corner pins. Next, 25 μl of BM cells were placed directly onto the filter sites. The chamber was then incubated for 2 h at 37°C in humid atmosphere with 5% CO_2_. Following incubation, the non-migrating cells on the origin side (top) of the filter were removed by gently wiping the filter with a tissue and the chamber was centrifuged at 200 g for 5 min at 20°C. The filter was removed and the number of neutrophils that migrated into the bottom compartment was determined by measuring MPO activity, as described above. The results were expressed as percent of neutrophils in relation of a standard curve of varying concentration of the original cells suspension.

### Eosinophil adhesion and chemotaxis assays

The number of eosinophils in BM was initially adjusted to 8 × 10^6^ cells/ml. Next, BM cells were incubated with SEA (1–30 ng/ml), IFN-γ (10 ng/ml) or sterile IMDM (control group) at 37°C, 5% CO_2_ for 30 min to 4 h. Adhesion and chemotaxis assays for eosinophils were performed as described below. Fifty microliter of BM cells were added to 96-well plates pre-coated with recombinant mouse VCAM-1 (2.5 μg/ml) or ICAM-1 (2.5 μg/ml) (Takeshita et al., 2015)_._ BM eosinophils were then stimulated with eotaxin (300 ng/ml) for 30 min, and adhesion was calculated by measuring the peroxidase (EPO) activity of adherent cells. To achieve this, 50 μL of EPO substrate (1 mM H_2_O_2_, 1 mM o-phenylenediamine and 0.1% Triton X-100 in Tris-buffer pH 8.0) were added to each well. After 30 min of incubation at room temperature, 25 μl of H_2_SO_4_ (4 M) were added to each well to stop the reaction and absorbance was measured at 490 nM with a microplate reader (Synergy H1 Hybrid Reader, Biotek, USA). Eosinophil adherence was calculated considering the optic density (OD) obtained in EPO assay divided by the number of eosinophils (EOS) added in each well (Conran et al., 2001).

For the eosinophil chemotaxis assays, a 96-multiwell chemotaxis chamber was used (Roviezzo et al., 2004). The wells in the microplate at the bottom compartment were filled with 29 μl of eotaxin (300 ng/ml). A polycarbonate filter (5 μm pore size) was positioned on the loaded microplate and hold in place with corner pins. Next, 25 μl of BM cells were placed directly onto the filter sites. The chamber was then incubated for 2 h at 37°C in humid atmosphere with 5% CO_2_. Following incubation, the non-migrating cells on the origin side (top) of the filter were removed by gently wiping the filter with a tissue and the chamber was centrifuged at 200 g for 5 min at 20°C. The filter was then removed and the number of cells that migrated into the bottom compartment was determined by measuring residual EPO activity, as described above. The results were expressed as % of eosinophils or neutrophils in relation of a standard curve of varying concentration of the original cells suspension.

### Measurements of intracellular Ca^2+^ levels in neutrophils and eosinophils

Intracellular Ca^2+^ levels were monitored in non-adherent BM cells (1 × 10^6^ cells/ml) loaded with a fluorogenic calcium-binding dye (FluoForte), according to a previous study (Takeshita et al., 2015). BM cells were incubated with SEA (10 ng/ml), and suspended in a medium containing Fluoforte (3 μM) for 45 min at room temperature protected from light. Thereafter, cell suspension was centrifuged at 400 g for 10 min. Aliquots of cell suspension (300 μl) were dispensed into 96 wells plates (Synergy H1 Hybrid Reader, Biotek, USA) equipped with a stirring device. To obtain total intracellular Ca^2+^ levels, the external Ca^2+^ concentration was adjusted to 1 mM with CaCl_2_, following equilibration for at least 30 s. Next, either IL-8 (200 ng/ml; for neutrophils) or eotaxin (300 ng/ml; for eosinophils) was added to induce cell activation. The Fluoforte fluorescence was monitored continuously with monochromator settings of 339 nm (excitation) and 500 mM (emission). The external influx of Ca^2+^ was calculated by subtracting the total Ca^2+^ levels from the internal storage mobilization. The intracellular calcium levels were calculated by use of a general formula: [Ca ^2+^]i = K_d_ × (F-F_min_)/(F_max_- F), were Kd = (389) is the Fluoforte dissociation constant, according to a previous study (Grynkiewicz et al., 1985). The F_max_ value was obtained with triton X-100 (0.1%) in the presence of saturating calcium (CaCl_2_ 1 mM), whereas F_min_ value was obtained with the calcium chelator EGTA (10 mM) plus Tris (20 mM; pH= 8).

### Effect of MHC class II blocking antibody

The effects of MHC class II blocking antibody on the adhesion and chemotactic responses of non-adherent BM cells after SEA or IFN-γ incubation were examined. Briefly, cells were incubated for 30 min at 37°C with mouse anti MHC class II blocking antibody (2 μg/ml) before addition of SEA, IFN-γ or IMDM. Next, BM cell adhesion or chemotaxis was assayed in the presence of either IL-8 (neutrophils) or eotaxin (eosinophils).

### Flow cytometry assays

Cells were incubated with SEA for 2 h (100 μL of 3 × 10^6^ cells/ml of PBS containing 0.5% BSA and 0.2% azide). Next, each cell sample was incubated another 20 min at 4°C with fluorescein isothiocyanate-conjugated anti-mouse MAC-1, phycoerythrin (PE)-conjugated anti-mouse VLA-4, or fluorescein isothiocyanate- conjugated anti-mouse LFA1-α. Subsequent analysis using a Becton-Dickinson FACSCalibur Cytometer (San Jose, CA) with CellQuest Pro software was done. MAC-1, VLA-4, and LF1-α fluorescent signals were acquired in the FL-2 and FL-1 channels, respectively. Fluorescent events considered for analysis were those that appeared on a gate positioned in the region expected to be enriched with granulocytes on SSC vs. FSC dot-plot. Acquisition of 10,000 events was performed for all samples. Prior to fluorescence acquisition, independent groups of cells incubated with either PE-conjugated mouse IgGk monoclonal isotype control, FITC rat IgG2a (for LFA1-α) or IgG2b (for MAC-1) isotype control were analyzed in order to discriminate unspecific fluorescence in FL-2 and FL-1 channels. Surface content of MAC-1, VLA-4, and LFA1α were expressed by the mean fluorescence obtained from the histogram.

### Measurement of IFN-γ in BM cell supernatant after sea incubation

The IFN-γ levels were measured in mouse BM cell supernatant using commercially available ELISA kits (Mouse DuoSet ELISA Development System), following the instructions of the manufacturer (R&D, Minneapolis, MN, USA).

### Statistical analysis

Data were presented as the mean values ± SEM and were analyzed by analysis of variance (ANOVA) for multiple comparisons followed by Bonferroni post-test, using a program package for statistical analysis (GraphPad software, version 5.00; San Diego, USA). *p* < 0.05 was considered significant.

## Results

### Inhibitory effects of sea on BM neutrophil adhesion and chemotaxis

**Figure 1:**
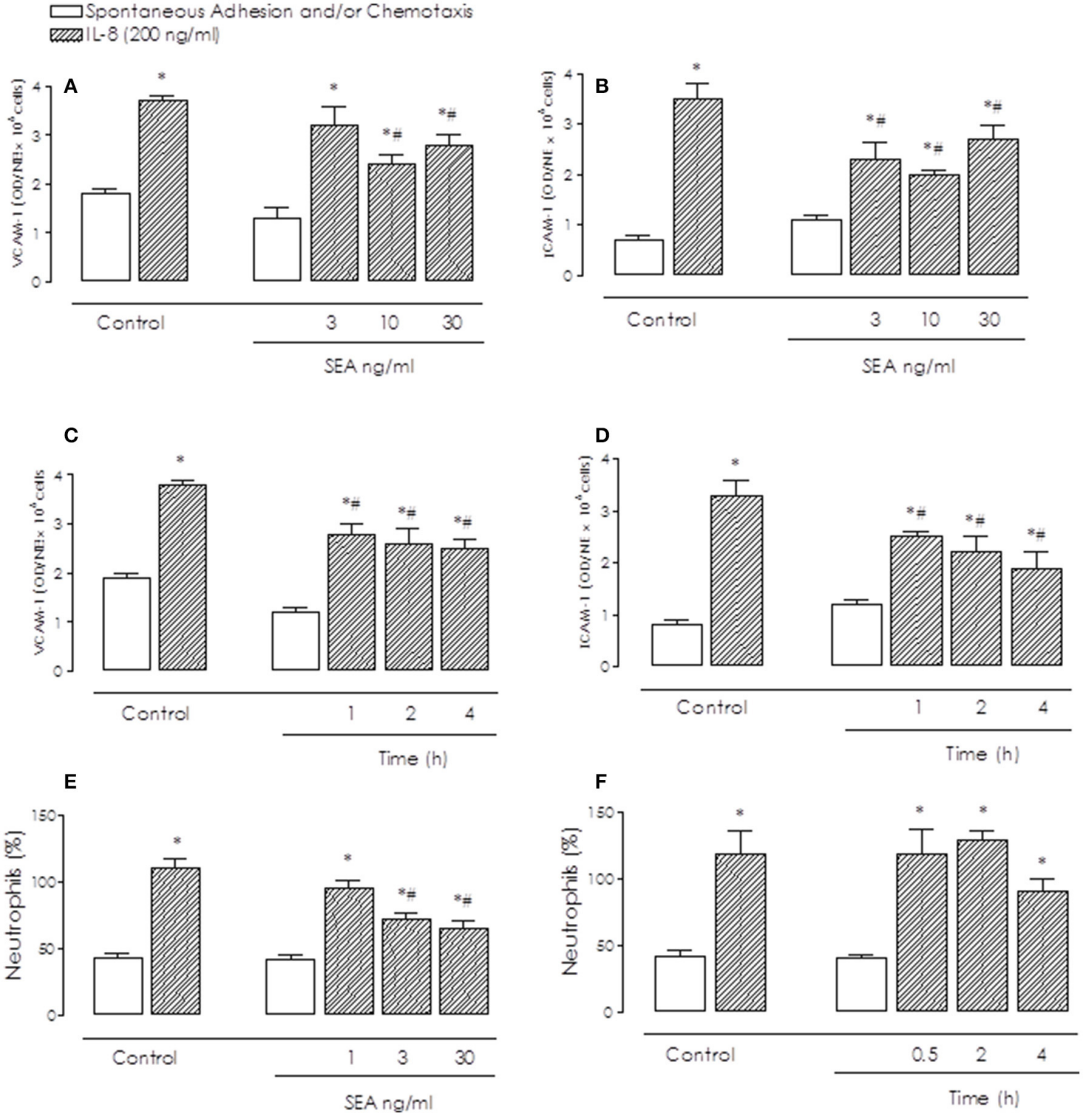
Inhibitory effects of Staphylococcal enterotoxin A (SEA) on IL-8-induced bone marrow (BM) neutrophil adhesion **(A–D)** and chemotaxis **(E,F)**. Neutrophils were incubated with SEA (1–30 ng/ml) or Iscove's modified Dulbecco's medium (Control) for 0.5 to 4 h, and placed to adhere on VCAM-1 or ICAM-1-coated plates or allowed to migrate in 96-multiwell chemotaxis chamber. Cells were stimulated or not with IL-8 (200 ng/ml). BM neutrophil adhesion and chemotaxis were quantified by MPO measurement. Results are mean values ± S.E.M from 5 mice for each group. ^*^p < 0.05 compared with spontaneous activity; #*p* < 0.05 compared with untreated cells (controls).

Activation of BM neutrophils with IL-8 (200 ng/ml) markedly enhanced the cell adhesion on both VCAM-1 and ICAM-1 pre-coated plates in comparison with non-stimulated cells (*P* < 0.05; Figures 1A–D). Prior incubation (2 h) of neutrophils with SEA (3–30 ng/ml) significantly (*P* < 0.05) decreased the IL-8-induced adhesion, as observed in both ICAM-1 and VCAM-1-coated plates (Figures 1A,B). Inhibition of IL-8-induced neutrophil adhesion by SEA was of the same magnitude at 1 to 4 h incubation (Figures 1C,D).

Prior incubation (2 h) of neutrophils with SEA (1–30 ng/ml) also significantly (*P* < 0.05) decreased the IL-8-induced chemotaxis compared with non-stimulated cells (Figure 1E). Incubation of neutrophils with SEA (10 ng/ml) for prolonged time periods (4 h) did not produce a further inhibition of cell chemotaxis (Figure 1F).

The spontaneous neutrophil adhesion and chemotaxis (in the absence of IL-8) were not significantly affected by incubation with SEA in any dose and incubation-time used.

MAC-1 and LFA 1α are key integrins responsible for the neutrophil functions (Furze and Rankin, 2008; Nourshargh and Alon, 2014). Therefore, the expressions of both proteins in BM cells were evaluated in the presence and the absence of SEA, using flow cytometry. The expressions of MAC-1 and LFA 1α were not significantly affected by SEA compared with untreated cells (Table 1).

**Table 1:** Effects of Staphylococcal enterotoxin A (SEA) on the MAC-1, VLA4, and LFA-1α expressions in bone marrow granulocytes.

	**Mean fluorescence intensity**		
	**MAC-1**	**VLA-4**	**LFA 1α**
**Control**	887.0 ± 40.5	492.4 ± 34.6	113.9 ± 14.9
**SEA**	1100.5 ± 99.2	500.9 ± 15.0	97.1 ± 9.7

*BM granulocytes were incubated with SEA (10 ng/ml) or Iscove's modified Dulbecco's medium (Control) for 2 h and gated for MAC-1, VLA-4 and LFA1-α population for the expression analysis of these molecules on flow cytometer. Results are mean values ± SEM from 5 mice for each group. p < 0.05 was considerate significantly*.

### Inhibitory effects of sea on BM eosinophil adhesion and chemotaxis

**Figure 2:**
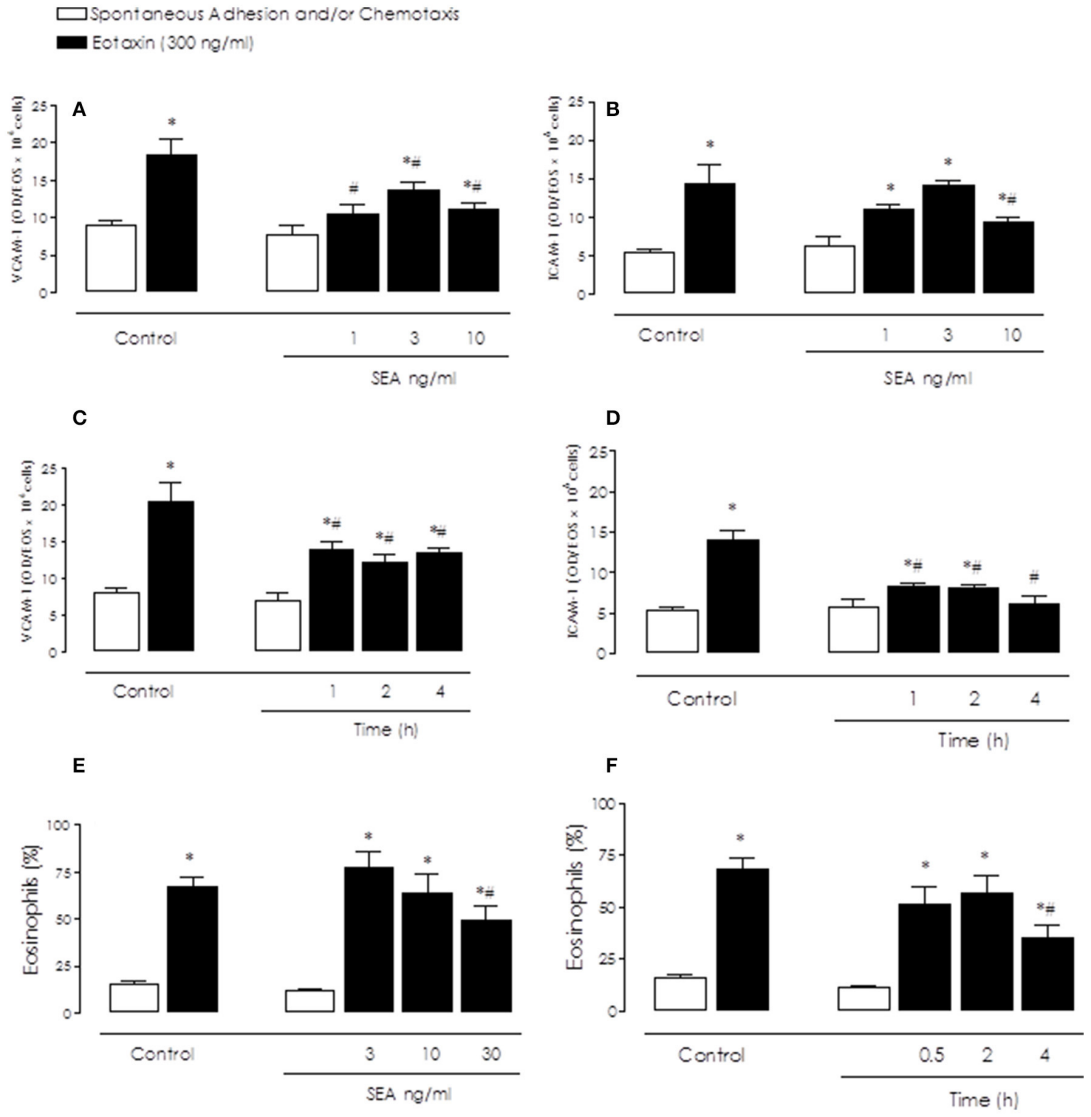
Inhibitory effects of Staphylococcal enterotoxin A (SEA) on eotaxin-induced bone marrow (BM) eosinophils adhesion **(A–D)** and chemotaxis **(E,F)**. BM eosinophils were incubated with SEA (1–30 ng/ml) or Iscove's modified Dulbecco's medium (Control) for 0.5–4 h, and placed to adhere on VCAM-1 or ICAM-1-coated plates or allowed to migrate in 96-multiwell chemotaxis chamber. Cells were stimulated or not with eotaxin (300 ng/ml). BM eosinophil adhesion and chemotaxis were quantified by EPO measurement. Results are mean values ± S.E.M from 5 mice for each group. ^*^p < 0.05 compared with spontaneous activities; #*p* < 0.05 compared with untreated cells.

Activation of BM eosinophils with eotaxin (300 ng/ml) markedly enhanced (*P* < 0.05) the cell adhesion in plates pre-coated with either VCAM-1 (Figures 2A,C) or ICAM-1 (Figures 2B,D) in comparison with non-stimulated cells (spontaneous activity). Prior incubation (2 h) of eosinophils with SEA (1–10 ng/ml) significantly (*P* < 0.05) reduced the eotaxin-induced adhesion in VCAM-1 and ICAM-1-coated plates in comparison with untreated cells (Control; Figures 2A,B). Maximal inhibition of eosinophil adhesion by SEA (10 ng/ml) on VCAM-1 and ICAM-1 was obtained at 1 h-incubation, maintaining the inhibition by 4 h-incubation (Figures 2C,D).

Similar inhibitory results were obtained in the chemotaxis assays (Figures 2E,F). Eosinophil chemotaxis was markedly increased (*P* < 0.05) in eotaxin-stimulated cells, which was significantly inhibited by SEA (30 ng/ml) (Figures 2E,F). The spontaneous eosinophil chemotaxis was not significantly affected by SEA in any dose and incubation-time used.

The spontaneous adhesion and chemotaxis of eosinophils were not significantly affected by SEA in any dose and incubation-time used.

VLA-4 is the main integrin responsible for the eosinophil functions (Stone et al., 2010; Nourshargh and Alon, 2014). We have therefore evaluated the expression of this protein in BM cells by using flow cytometry. The expression of VLA-4 was not significantly affected by SEA compared with untreated cells (Table 1).

### Intracellular Ca^2+^ mobilization in BM cells

In BM cells activated with IL-8 (200 ng/ml) the intracellular Ca^2+^ levels was significantly increased (*P* < 0.05) compared to basal conditions (Table 2). Prior incubation with SEA significantly inhibited the intracellular Ca^2+^ mobilization in IL-8-activated cells. Similarly, in BM cells activated with eotaxin (300 ng/ml), the intracellular Ca^2+^ levels significantly increased (*P* < 0.05) compared to basal conditions, which was markedly reduced by incubation with SEA (Table 2).

**Table 2:** Effects of Staphylococcal enterotoxin A (SEA) on intracellular Ca^2+^ mobilization in bone marrow granulocytes.

	**Intracellular Ca^2+^ mobilization (nM)**		
	**Basal**	**IL-8**	**Eotaxin**
**Control**	187.4 ± 17.5	537.0 ± 46.0^*^	320.9 ± 34.2^*^
**SEA**	194.9 ± 34.8	391.0 ± 46.0#	231.0 ± 37.7#

BM cells were incubated with SEA (10 ng/ml) and suspended in a medium containing Fluoforte (3 μM, 45 min), after which cells were stimulated with eotaxin (300 ng/ml) or IL-8 (200 ng/ml). Results are mean values ± SEM from 8 mice for each group.

* p < 0.05 compared with basal values.

# *p < 0.05 compared with respective IL-8 or eotaxin values*.

### Effect of anti-MHC class II antibody on BM cell adhesion and chemotaxis

**Figure 3:**
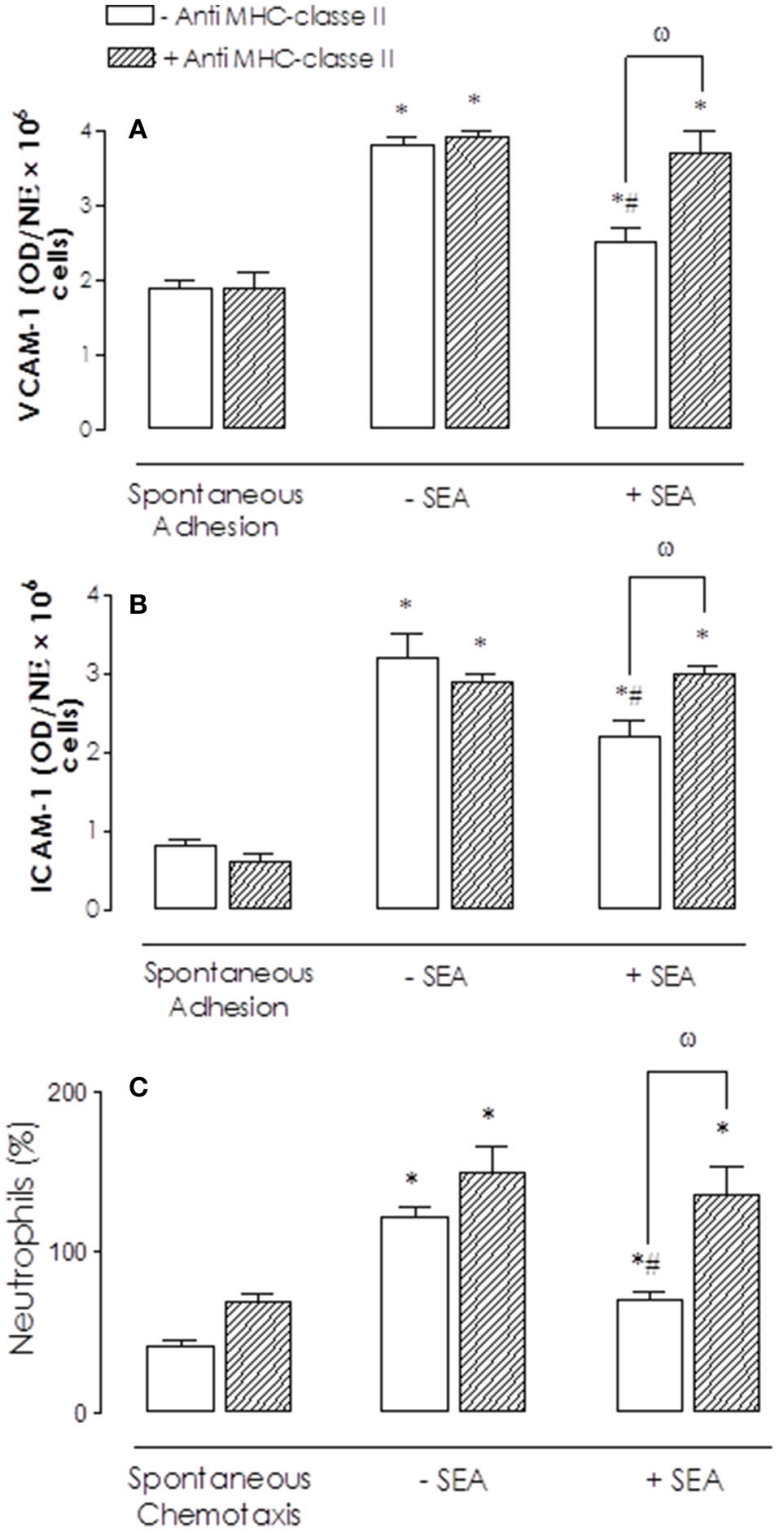
Effect of anti-MHC class II antibody on SEA-mediated inhibition of bone marrow (BM) neutrophil adhesion and chemotaxis. Neutrophils were incubated with anti-MHC class II antibody (2 μg/ml; 30 min) before addition of SEA (10 ng/ml, 2 h **A,B**; 30 ng/ml, 2 h **C**). IL-8 (200 ng/ml)-activated neutrophils were placed to adhere on VCAM-1 or ICAM-1-coated plates or allowed to migrate in 96-multiwell chemotaxis chamber. Neutrophil adhesion and chemotaxis were revealed by MPO measurements. Results are mean values ± S.E.M from 5 mice for each group. ^*^p < 0.05 compared with spontaneous activities; #*p* < 0.05 compared with untreated cells (controls); ω *p* < 0.05 compared with anti-MHC class II antibody in untreated cells (absence of SEA).

BM cells were incubated with anti-MHC class II antibody (2 μg/ml; 30 min, 37°C, 5% of CO_2_) before SEA incubation (10 ng/ml, 2 h). Anti-MHC class II antibody prevented he reductions by SEA of IL-8-induced adhesion (Figures 3A,B) and chemotaxis (Figures 3C) in comparison to non-treated cells.

**Figure 4:**
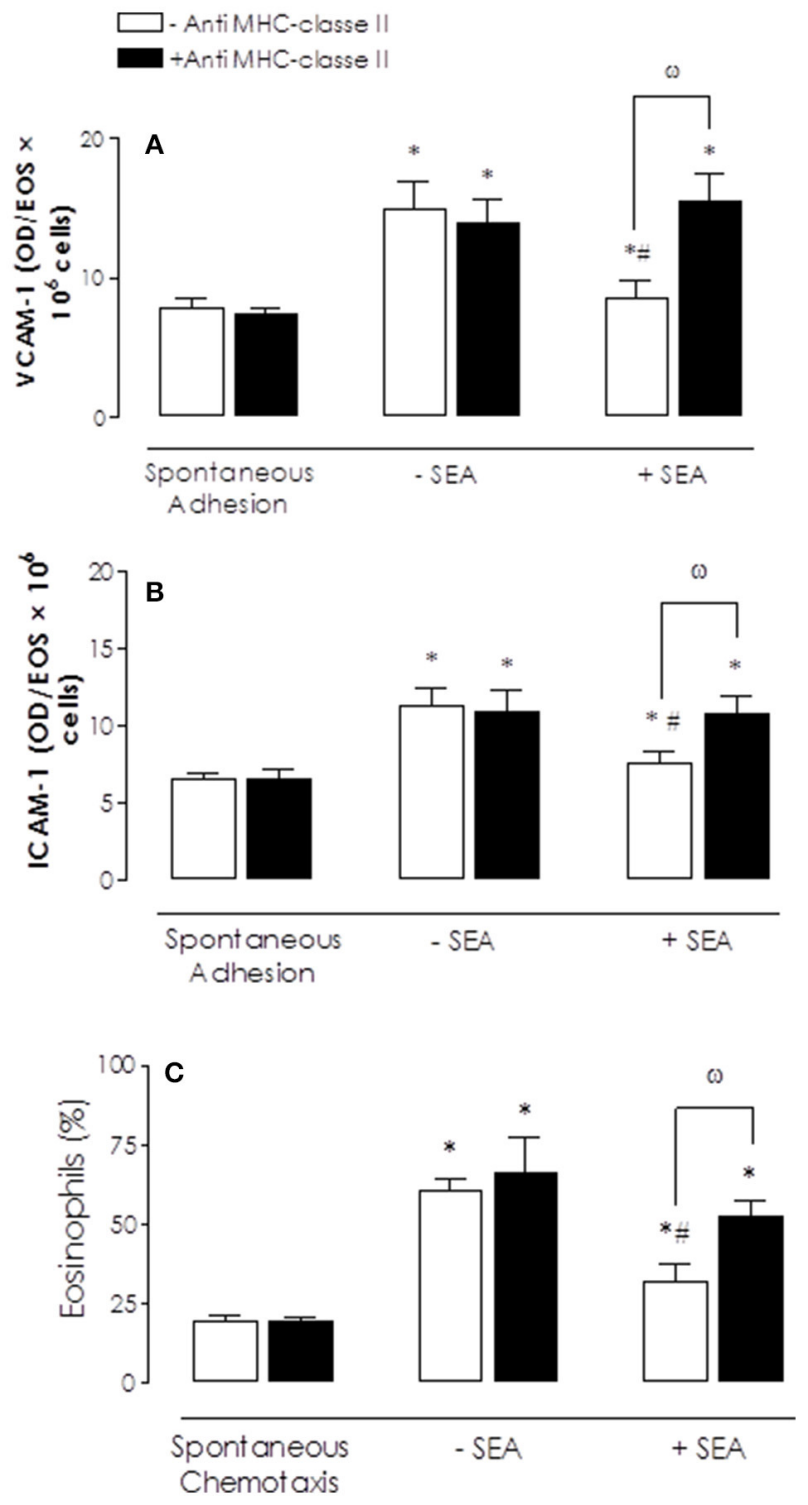
Effect of anti-MHC class II antibody on SEA-mediated inhibition on bone marrow (BM) eosinophil adhesion and chemotaxis. Eosinophils were incubated with anti-MHC class II antibody (2 μg/ml; 30 min) before addition of SEA (10 ng/ml, 2 h **A,B**; 30 ng/ml, 2 h; **C**). Eotaxin (300 ng/ml)-activated cells were placed to adhere on VCAM-1 or ICAM-1-coated plates or allowed to migrate in 96-multiwell chemotaxis chamber. Eosinophil adhesion and chemotaxis were revealed by EPO measurement. Results are mean values ± S.E.M from 5 mice for each group. ^*^p < 0.05 compared with spontaneous activities; #*p* < 0.05 compared with untreated cells (controls); ω *p* < 0.05 compared with anti-MHC class II antibody in untreated cells (absence of SEA).

Likewise, pre-treatment with anti-MHC class II antibody fully prevented the reduction by SEA of the eotaxin-induced eosinophil adhesion (Figures 4A,B) and chemotaxis (Figure 4C) in comparison to non-treated cells.

### Inhibitory effects of IFN-γ on BM cell adhesion and chemotaxis

**Figure 5:**
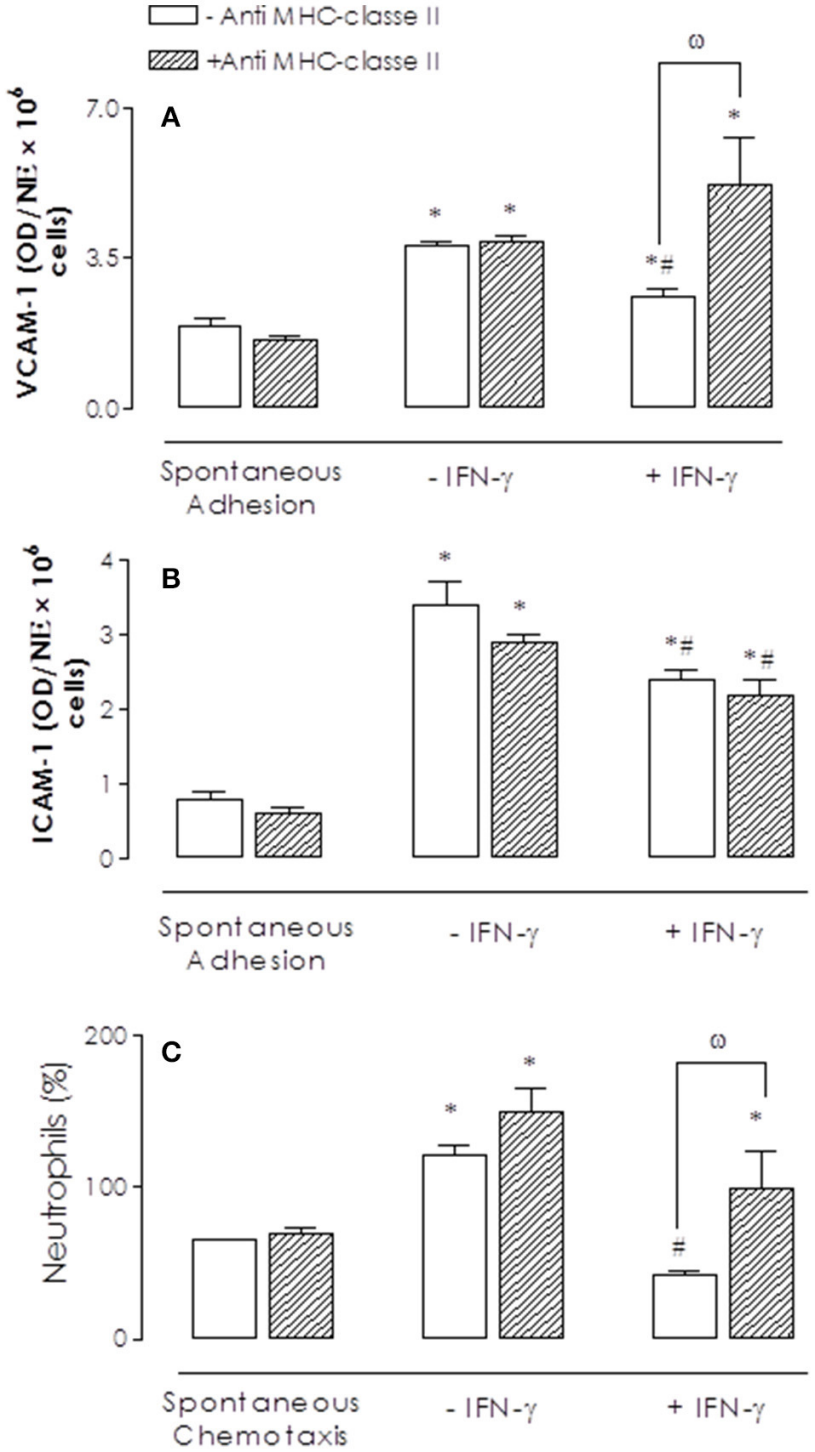
Inhibitory effects of interferon-γ (IFN-γ) on IL-8-induced bone marrow (BM) neutrophils adhesion and/or chemotaxis and reversal by anti-MHC class II antibody. Neutrophils were incubated with anti-MHC class II antibody (2 μg/ml; 30 min) before addition of IFN-γ (10 ng/ml, 2 h). IL-8 (200 ng/ml)-activated neutrophils were placed to adhere on VCAM-1 or ICAM-1-coated plates **(A–B)** or allowed to migrate in 96-multiwell chemotaxis chamber **(C)**. Neutrophil adhesion and chemotaxis were revealed by MPO measurements. Results are mean values ± S.E.M from five mice for each group. ^*^p < 0.05 compared with activities; ^#^*p* < 0.05 compared with untreated cells (controls); ^ω^*p* < 0.05 compared with anti-MHC class II antibody in untreated cells (absence of IFN-γ).

The levels of INF-γ was greater in the cell supernatant after SEA stimulation (10 ng/ml, 2 h; *P* < 0.05) when compared with control group (30.6 ± 6.2 and 104.3 ± 28.3 pg/mg protein for control and SEA, respectively). We next evaluated the effects of exogenous INF-γ addition on adhesion and chemotaxis of neutrophils and eosinophils. Figure 5 shows that incubation of neutrophils with IFN-γ (10 ng/ml) significantly reduced (*P* < 0.05) the IL-8-induced adhesion on VCAM-1 and ICAM-1 coated plates. Prior incubation with anti-MHC class II antibody prevented the reduced IFN-γ-induced adhesion on VCAM-1 (but not ICAM-1)-coated plates (*P* < 0.05) (Figures 5A,B). Incubation of neutrophils with IFN-γ also significantly reduced the IL-8-induced chemotaxis, which was prevented by prior treatment with anti-MHC class II antibody (Figure 5C).

**Figure 6:**
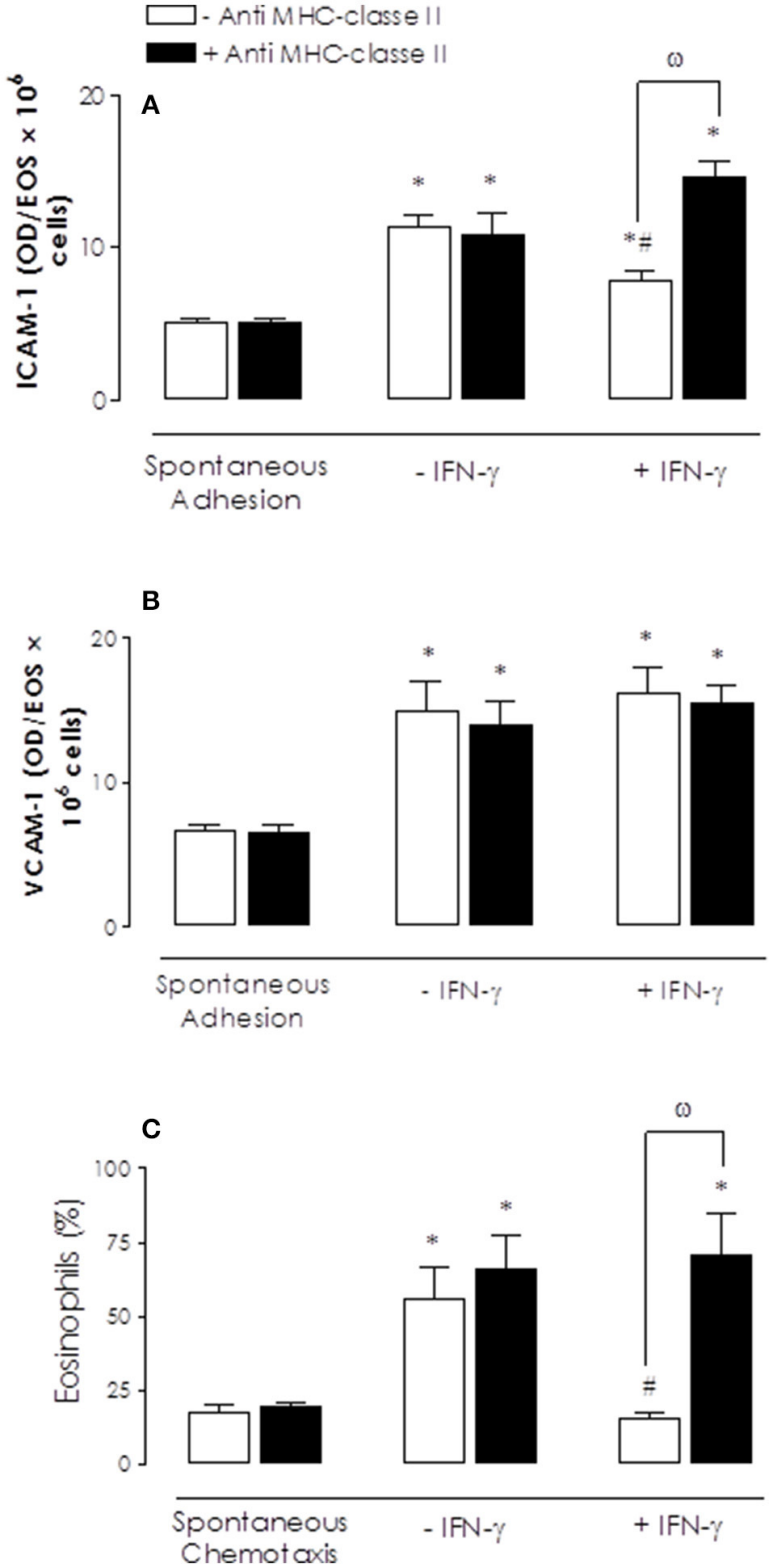
Inhibitory effects of interferon-γ (IFN-γ) on eotaxin-induced bone marrow (BM) eosinophils adhesion and/or chemotaxis and reversal by anti-MHC class II antibody. Eosinophils were previous incubated with anti-MHC class II antibody (2 μg/ml; 30 min) before addition of IFN-γ (10 ng/ml, 2 h). Eotaxin (300 ng/ml)-activated cells were placed to adhere on VCAM-1 or ICAM-1-coated plates **(A–B)** or allowed to migrate in 96-multiwell chemotaxis chamber (**C**). Eosinophil adhesion and chemotaxis were revealed by EPO measurement. Results are mean values ± S.E.M from five mice for each group. ^*^p < 0.05 compared with spontaneous activities; ^#^*p* < 0.05 compared with (controls) and ^ω^*p* < 0.05 compared with anti-MHC class II antibody in untreated cells (absence of IFN-γ).

Incubation of eosinophils with IFN-γ (10 ng/ml, 2 h) significantly reduced the eotaxin-induced adhesion on ICAM-1 (but not VCAM-1), as well as on cell chemotaxis (Figure 6). These reductions by IFN were fully restored (*P* < 0.05) by prior incubation with anti-MHC class II antibody.

## Discussion

Our present study demonstrates that SEA markedly reduces the *in vitro* chemotactic and adhesive responses of activated BM neutrophils and eosinophils, an effect associated with reduced intracellular Ca^2+^ levels. Prior incubation of these cells with anti-MHC class II blocking antibody prevented the reduced adhesion and chemotaxis by SEA, indicating a major role for MHC class II to produce cell inhibition.

Neutrophil and eosinophil adhesion to endothelium is a crucial step to the mobilization of these cells to inflammatory tissues (Timmerman et al., 2016). In neutrophils, this process is mediated by MAC-1 (αMβ2; CD11/CD18) and by the integrin lymphocyte antigen function-1 (LFA-1α; αLβ2, CD11a/CD18), which interacts with ICAM-1 and VCAM-1 immunoglobulins in endothelium, respectively (Furze and Rankin, 2008; Nourshargh and Alon, 2014). In eosinophils, the firm adhesion of these cells to endothelium takes place mainly via VLA-4 (CD49d/CD29; α4β1) (Park and Bochner, 2010; Stone et al., 2010; Nourshargh and Alon, 2014). In addition to adhesion molecules, leukocyte trafficking to inflammatory site requires the induced release of a wide range of inflammatory chemokines (Wedepohl et al., 2012; Nourshargh and Alon, 2014). One of the major mediators of neutrophil recruitment and activation during inflammatory conditions is the CXC chemokine IL-8, which acts on neutrophils via two distinct types of receptor, CXC chemokine receptor 1 (CXCR1) and CXCR2. Evidence indicates that CXCR1 could have a dominant role in mediating IL-8 chemotaxis of neutrophils (Nasser et al., 2007; Admyre et al., 2014). Several inflammatory conditions and infectious diseases also comprise the selective eosinophil chemotaxis and transendothelial migration mediated by the CC-chemokines eotaxin-1, eotaxin-2 and eotaxin-3 (CCL11, CCL24, and CCL26, respectively) via the eotaxin receptor CCR3 (Ponath et al., 1996).

There is compelling evidence indicating that neutrophil function during severe sepsis is substantially dysregulated, resulting in reduced migration to infectious site hence producing an inadequate antimicrobial response (Alves-Filho et al., 2010; Xu et al., 2014). Impaired neutrophil chemotactic responses and changes in neutrophil function in sepsis directly correlates with increased morbidity and mortality (Danikas et al., 2008). Accordingly, in a mouse sepsis model induced by *S. aureus*, circulating neutrophils failed to migrate in response to the chemoattractants fMLP and leukotriene B_4_ (Crosara-Alberto et al., 2002). Moreover, the Staphylococcal superantigen-like protein 5 (SSL5) inhibits rolling and adhesion of neutrophils to P-selectin and human endothelial cells (Itoh et al., 2010). A chemotaxis inhibitory protein isolated from *S. aureus* (CHIPS) was also shown to inhibit the fMLP- and C5a-induced responses in neutrophils (Postma et al., 2005). In addition, the reduced counts of circulating eosinophils seem to constitute a good diagnostic marker to identify sepsis conditions and discriminating its severity (Abidi et al., 2008).

In our study, incubation of neutrophils with SEA at varying concentrations and time-periods markedly inhibited the IL-8-induced BM neutrophil adhesion and chemotaxis, which was accompanied by reduced intracellular Ca^2+^ levels. Similar data were obtained with eotaxin-induced eosinophil adhesion and chemotaxis. However, incubation with SEA did not modify the expression of MAC-1 and LFA-1α on neutrophils (or VLA-4 on eosinophils), suggesting that down-regulation of these integrins do not explain the inhibitory effects of SEA on neutrophils and/or eosinophil activities. It is well established that Staphylococcal enterotoxins such as SEA directly bind to MHC class II molecules on antigen-presenting cells, generating a variety of inflammatory and immune responses (Fraser, 2011). Our findings show that prior incubation of neutrophils and eosinophils with anti-MHC antibody fully prevented the inhibition by SEA on adhesion and chemotaxis of neutrophils and eosinophils, indicating a crucial role for MHC class II in triggering cell inhibition by this enterotoxin. MHC class II molecules are classically described in APC (Afridi et al., 2016), but recent studies show its presence in human neutrophils (Pliyev et al., 2014) and eosinophils (Akuthota et al., 2012).

IFN-γ is a potent pro-inflammatory cytokine during the development of innate and adaptive immune reaction in microbial infections (Swindle et al., 2015). IFN-γ is predominantly produced by T cells and natural killer (NK) cells and orchestrates a vast array of pathological responses (McNab et al., 2015). Evidence shows that IFN-γ acts as a suppressor of hematopoiesis and BM failure resulting from chronic inflammation (de Bruin et al., 2014). IFN-γ has also been considered the main mediator of damage and lethal effects after Staphylococcal enterotoxin exposure in mice (Rao et al., 2014). In addition, the pathological effects of SEA in the lungs are dependent on IFN-γ (Muralimohan et al., 2008). High levels of IFN-γ in lymph nodes are found in SEA-exposed mice (Kuroda-Morimoto et al., 2010) that are associated with increased expression of endothelial adhesion molecules (Zhang et al., 2011). IFN-γ is also described to induce MHC class II overexpression in macrophages (Hang et al., 2011) and epithelium (Thelemann et al., 2014). In our study, higher levels of IFN-γ were found in the BM cell supernatant of SEA-treated cells. We therefore tested the hypothesis that IFN-γ release via MHC class II mediates the inhibitory effects of SEA. Prior incubation of neutrophils and eosinophils with IFN-γ significantly reduced the *in vitro* chemotactic and adhesive properties of these cells, which was prevented by incubation with anti-MHC class II antibody in a similar fashion of SEA. These data support the view that SEA exposure increase IFN-γ levels, which in turn activate MHC class II in leukocyte membrane, culminating in the inhibition of important cell effector functions such as chemotaxis and adhesion. We may consider, however, that BM granulocytes also include some contamination with lymphocyte (30%), which is an important source of IFN-γ.

## Conclusion

In summary, our present findings show that SEA exposure activates MHC class II in BM cells, leading in turn to inhibition of adhesion and chemotaxis of both neutrophils and eosinophils. This inhibitory mechanism by SEA may explain the impaired adhesion and recruitment of leukocytes to infection sites in pathological conditions such as sepsis. Additionally, in our study, SEA increased the levels of IFN-γ in supernatant of BM cells. Incubation of BM cells with IFN-γ also significantly reduced the adhesion and chemotaxis of neutrophils and eosinophils via MHC class II activation. It is thus possible that leukocyte inhibition by SEA takes place by IFN-γ acting on MHC class molecule. This strategy to impair granulocyte locomotion to infection sites in the course of sepsis may represent an opportunity for the pathogen to establish residence and begin its systemic spread.

## Author contributions

ID and EA conceived the research and wrote the manuscript, AC-N contributed the materials, AF-D and AP-T performed the most part of the experiments, GA performed the flow cytometry assays. All authors edited and approved the manuscript.

### Conflict of interest statement

The authors declare that the research was conducted in the absence of any commercial or financial relationships that could be construed as a potential conflict of interest.
